# Supplementary material for: Isolation and Bioassay of a New Terminalone A from Terminalia arjuna
Source: Molecules. 2023 Jan 19;28(3):1015. doi: 10.3390/molecules28031015 (PMC9919985; doi:10.3390/molecules28031015)
Supplement: Supplementary file 1 [file molecules-28-01015-s001.zip › molecules-2144813-supplementary.pdf]

Supporting Information

Isolation and Bioassay of a New Terminalone A from *Terminalia arjuna*

Bushra[1], Khalil ur Rehman[1]*, Dilfaraz Khan[1], Abdulrahman A. Almehizia[2], Ahmed M. Naglah[2], Asma S. Al-Wasidi[3], Moamen S. Refat[4], Mohamed Y. El-Sayed[5], Hamid Ullah[6], Shafiullah Khan[1]*

[1]Institute of Chemical Sciences, Gomal University, Dera Ismail Khan, KPK, Pakistan

[2]Drug Exploration and Development Chair (DEDC), Department of Pharmaceutical Chemistry, College of Pharmacy, King Saud University, Riyadh 11451, Saudi Arabia

[3]Department of Chemistry, College of Science, Princess Nourah bint Abdulrahman University, Riyadh 11671, Saudi Arabia

[4]Department of Chemistry, College of Science, Taif University, P.O. Box 11099, Taif 21944, Saudi Arabia

[5]Chemistry Department, College of Science, Jouf University, P.O. Box: 2014, Sakaka, Saudi Arabia

[6]Department of Chemistry, Balochistan University of Information Technology, Engineering and Management Sciences, Quetta 87300, Pakistan

*__Corresponding author Email Address:__ khalilrehmanph.d@gmail.com,

s.khan@gu.edu.pk;

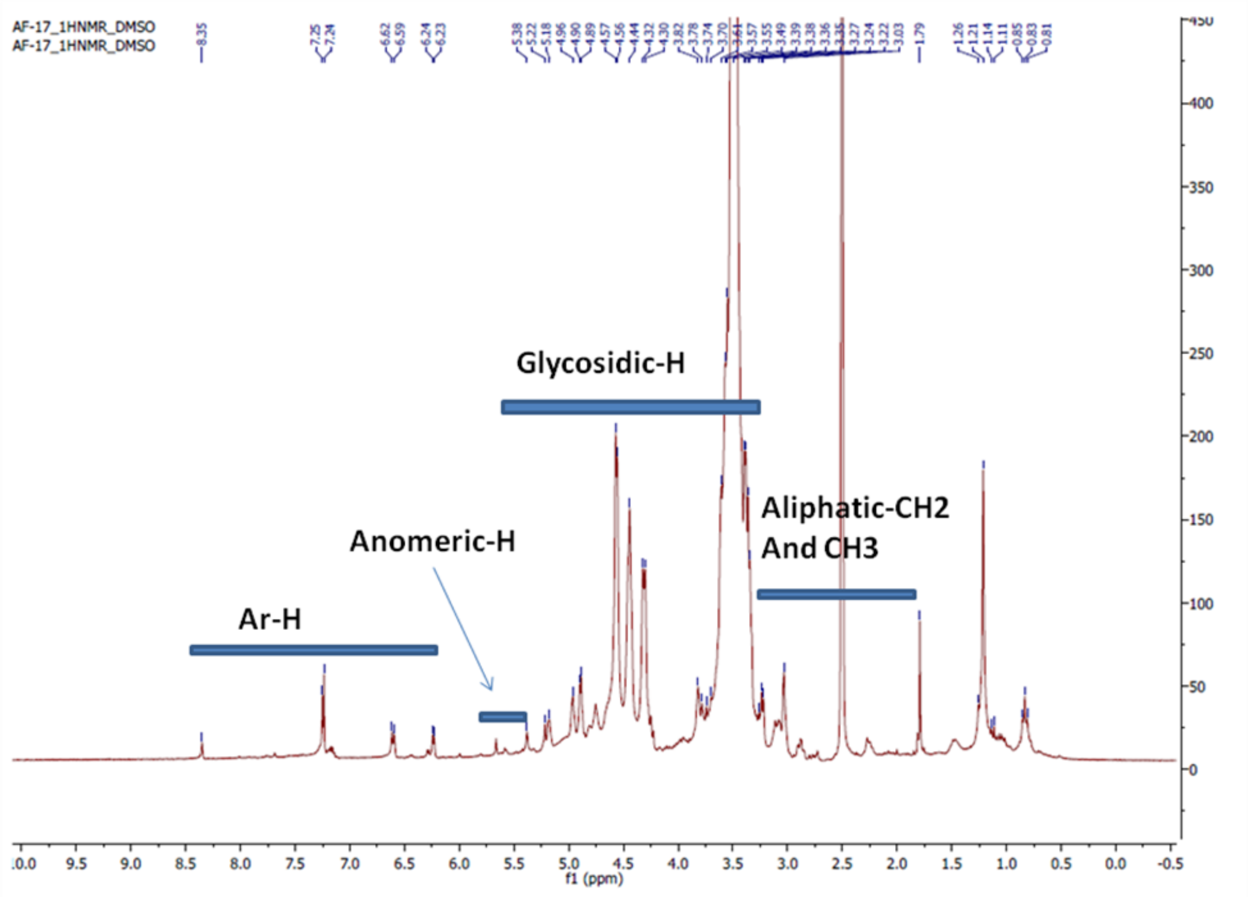

Figure S1: ^{1}H NMR spectrum of compound **1**

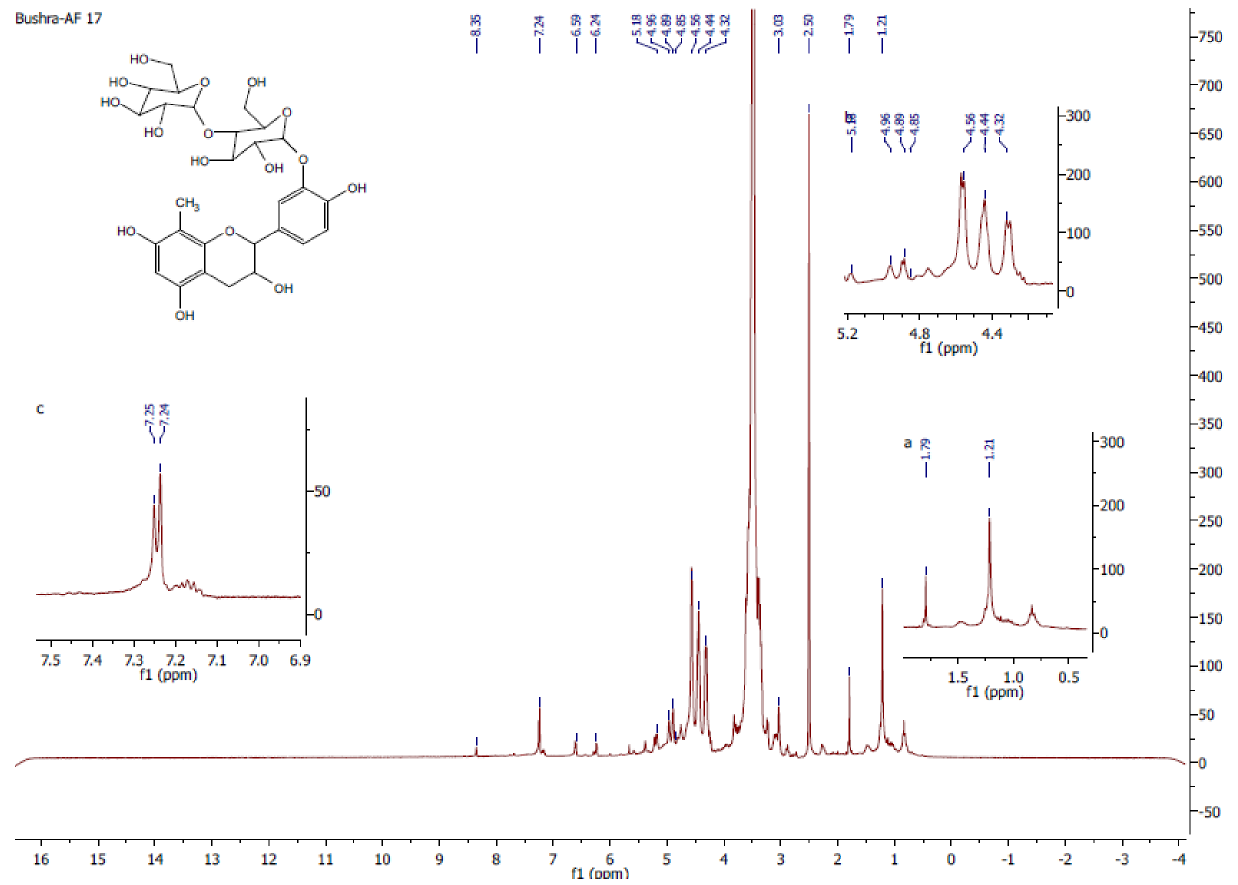

Figure S2: Expended ^{1}H NMR spectrum of compound **1**

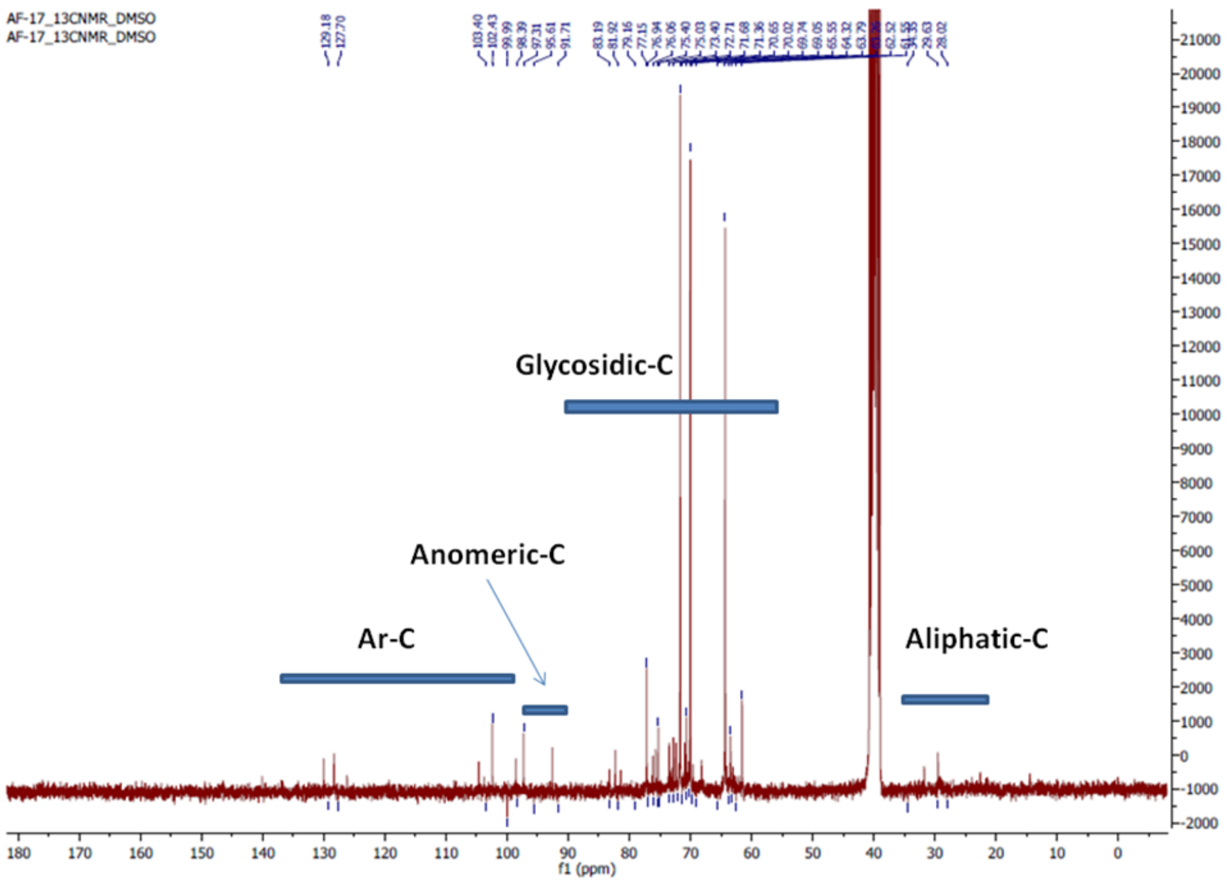

Figure S3: ^{13}C NMR spectrum of compound **1**
